# Structural Basis for the Mechanism of ATP-Dependent Acetone Carboxylation

**DOI:** 10.1038/s41598-017-06973-8

**Published:** 2017-08-03

**Authors:** Florence Mus, Brian J. Eilers, Alexander B. Alleman, Burak V. Kabasakal, Jennifer N. Wells, James W. Murray, Boguslaw P. Nocek, Jennifer L. DuBois, John W. Peters

**Affiliations:** 1 Insitutite of Biological Chemistry, Washington State University, Pullman, WA 99164 USA; 2 Department of Chemistry and Biochemistry, Montana State University, Bozeman, MT 59717 USA; 3 Department of Life Sciences, Imperial College, London, SW7 2AZ UK; 4 Structural Biology Center, Argonne National Laboratory, Argonne, IL 60439 USA

## Abstract

Microorganisms use carboxylase enzymes to form new carbon-carbon bonds by introducing carbon dioxide gas (CO_2_) or its hydrated form, bicarbonate (HCO_3_^−^), into target molecules. Acetone carboxylases (ACs) catalyze the conversion of substrates acetone and HCO_3_^−^ to form the product acetoacetate. Many bicarbonate-incorporating carboxylases rely on the organic cofactor biotin for the activation of bicarbonate. ACs contain metal ions but not organic cofactors, and use ATP to activate substrates through phosphorylation. How the enzyme coordinates these phosphorylation events and new C-C bond formation in the absence of biotin has remained a mystery since these enzymes were discovered. The first structural rationale for acetone carboxylation is presented here, focusing on the 360 kDa (αβγ)_2_ heterohexameric AC from *Xanthobacter autotrophicus* in the ligand-free, AMP-bound, and acetate coordinated states. These structures suggest successive steps in a catalytic cycle revealing that AC undergoes large conformational changes coupled to substrate activation by ATP to perform C-C bond ligation at a distant Mn center. These results illustrate a new chemical strategy for the conversion of CO_2_ into biomass, a process of great significance to the global carbon cycle.

## Introduction

Carboxylases are enzymes that catalyze the incorporation of CO_2_ into organic substrates. Assimilatory carboxylases use the carboxylation reaction to directly transform diverse carbon sources into central metabolites as part of autotrophic (photosynthetic) or heterotrophic metabolic pathways. The latter pathways are essential for the biological assimilation of often chemically intransigent, poorly activated organic compounds^1^. Most enzymatic carboxylation reactions follow the same mechanistic principle: nucleophilic activation of substrates and electrophilic activation of CO_2_^2^. However, the stepwise mechanisms of carboxylation reactions differ in essential ways with respect to co-substrate, co-factor or metal requirements. Knowledge of these mechanisms provides the basis for an increased fundamental understanding of carboxylation chemistry, and contributes to future strategies for CO_2_ capture. These in turn may mitigate the effects of increasing concentrations of CO_2_ on the global climate^3^.

**Figure 1:**
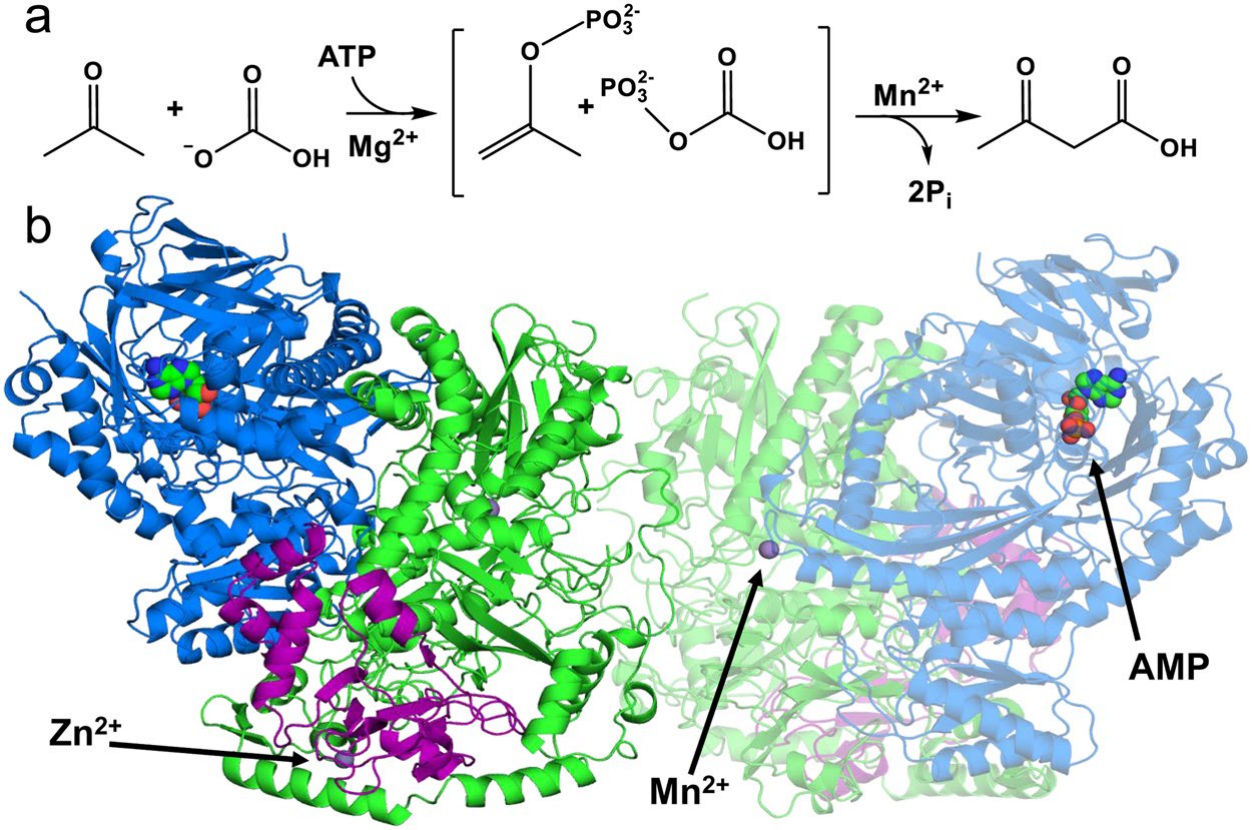
Overall reaction scheme and crystal structure of AMP bound AC. (**a**) Reaction of ACs. The sequential phosphorylation of the products acetone and then bicarbonate by the γ then β phosphates from ATP, respectively, creates the highly reactive intermediates phosphoenolacetone and carboxyphosphate. These are proposed to react together at the Mn^2+^ active site to create acetoacetate and two molecules of inorganic phosphate (Pi). (**b**) The overall structure of the (αβγ)_2_ heterohexameric AC enzyme is shown: α subunits (green), β subunits (blue), γ subunits (violet). One monomer is transparent to indicate the dimer interface as well as the nucleotide binding site. Mn^2+^, Zn^2+^ and AMP binding sites are indicated with arrows.

Acetone carboxylases are assimilatory carboxylases that catalyze the conversion of substrates acetone and HCO_3_^−^ to form the product acetoacetate (Fig. 1a) allowing bacteria to incorporate this small, volatile and environmentally toxic ketone into biomass. In general, bicarbonate-dependent carboxylases catalyze the net dehydration of H_2_CO_3_, retaining CO_2_ as a biotin adduct^4^. However, ACs purified from multiple bacterial sources have been shown to be free of biotin or any other organic cofactor, instead containing quantities of manganese, zinc, and iron within a heteromultimeric protein complex^5–8^. These carboxylases were also shown to convert ATP to AMP and two inorganic phosphate anions, suggesting that they catalyze the phosphorylation-dependent activation of both carbon substrates from a single nucleotide^9^.

This reaction sets ACs apart from the phylogenetically related acetophenone carboxylases (APCs). APC hydrolyses two ATP to ADP in order to activate acetophenone and bicarbonate^10^. The AC β subunit and APC α subunits share homology and both possess nucleotide-binding sites. The structure of APC was recently determined, revealing that the two MgATP binding sites that are the proposed sites for the activation of acetophenone and bicarbonate are separated by ~50 Å. A large conformational shift was proposed to bring the two phosphorylated intermediates in closer proximity for catalysis^11^.

Distinct from APC, AC from *Xanthobacter autotrophicus* Py2 has been shown to activate both acetone and bicarbonate with a single ATP, presumably using both γ and β phosphates in a sequential fashion, for a ligation reaction at a Mn-containing catalytic site^9^. The lack of structural information on ACs has been a significant impediment in formulating plausible mechanistic hypotheses. Here we present the first x-ray crystal structures of the 360 kDa (αβγ)_2_ heterohexameric AC. Surprisingly, the ATP binding site and the catalytically essential Mn cofactor, long assumed to be adjacent to one another on the basis of previous spectroscopic studies^12^, are separated by ~40 Å. A series of structures in the ligand free, AMP-bound, and acetate-coordinated states, which we represent as approximate mechanistically relevant states, allows the inference of a new mechanism for enzyme-mediated CO_2_ capture and functionalization.

## Results and Discussion

Like many carboxylases, AC is a heteromultimeric enzyme complex. Its architecture consists of two heterotrimeric αβγ subunits joined by the interacting α-subunits to form a dimeric core (Figs 1b and S1). The α subunit (75 kDa) shares a large interface with the β subunit (85 kDa). The γ subunit (20 kDa) interacts mostly with the α subunit and shares a small contact with the β subunit through a helix at the carboxyl end of the γ subunit. This interaction area between all three subunits creates a cleft on the solvent surface. AC’s α subunit shows high structural similarity to APCβ and contains similar internal folding domains, including the α/β interface. This interface is anchored by the large helix-2 on the α-subunit that leads into the polyproline II-like helix bundle similar to what is observed in APC^11^. The β subunit interface is made up of two α/β sandwich-like domains with exposed α-helices that interact directly with helix-2 from the α subunit. The nucleotide-binding β subunit has low structural similarity with kinases such as glycerol kinase (PDB: 3FLC, DALI-Z score 10.9), pantothenate kinase (PDB: 3BF3, DALI-Z score 12.2), and L-rhamnulose kinase (PDB: 2CGL, DALI-Z score 9.1), which share similar nucleotide-binding pockets^13–15^. The β subunit also shares nucleotide binding residues with mutually homologous subunits APCα and APCα’ (PDB: 5L9W, DALI-Z score 36.5 and 46.4 respectively). The γ-subunits, which bear homology to nucleotide binding Yippee-like domains, contain conserved cysteine residues (Cys74, Cys76, Cys124, Cys127) that form a 4-coordinate Zn binding site^16^. The role of the γ-subunit is not clear, and the Zn ion is not predicted, from prior data, to have a catalytic role. *X. autotrophicus* Py2 AC (XaAC) differs from that reported for *Aromatoleum aromaticum* (AaAC). XaAC has been reported as having 1.3 mol Mn and 0.7 mol Fe per mol of enzyme and AaAC has been reported to have significantly different metal content with no Mn and 2.2 mol Fe per mol of enzyme^8^. In addition, the stoichiometry for ATP consumption reported is also different with XaAC requiring only 1 ATP for acetoacetate formation and AaAC requiring 2 ATP^5, 8^. These differences are surprising given the high level of sequence conservation of the two enzymes. However, the observed differences in metal content in ACs purified from different sources are likely related to the differences in stoichiometry for ATP. Although the presence of Fe has been observed in previous studies of *X. autotrophicus* Py2 AC (0.7 Fe/(αβγ)_2_)^5^, K-edge anomalous difference data were not consistent with Fe in the structures presented here (see Supplementary Data, Table S2). The lack of Fe in the crystal data and previous studies by Boyd *et al*.^12^ suggest that Fe may associate with the enzyme in the absence of manganese but is not a catalytically effective subsitute^12^. Both the recombinant and native *X. autotrophicus* Py2 acetone carboxylases used for crystallization studies actively catalyzed the ATP- and HCO_3_^−^ -dependent carboxylation of acetone to acetoacetate at comparable levels to and with an overall stoichiometry similar to that described previously^5^.

**Figure 2:**
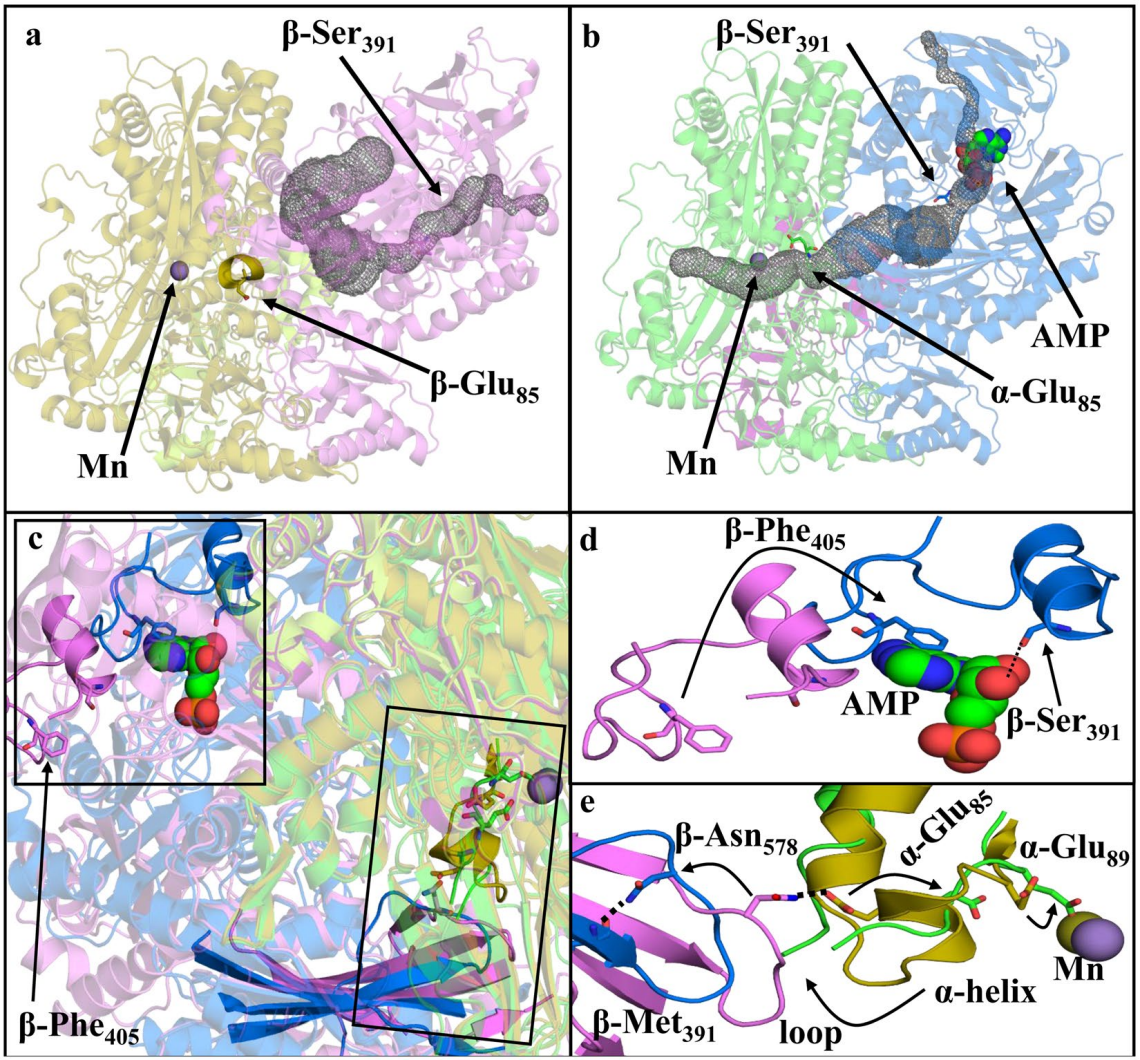
Conformational shift upon nucleotide binding. (**a**) Ligand-free structure (α subunits: olive; β subunits: violet; γ subunits: limon) showing a substrate channel (grey) linking the nucleotide binding site to solvent and allowing ATP and substrates to enter. Access to the Mn site is closed off in this structure by an Mn proximal α-helix that prevents the substrate channel from reaching the Mn active site. (**b**) AMP-bound structure (α subunits: green, β subunits: blue, γ subunits: pink) showing an opening of an internal channel (grey) linking the nucleotide binding site to the Mn binding site. The Mn proximal helix becomes a disordered loop region when AMP is bound, permitting access to the Mn. (**c**) Superposition of the ligand-free structure on the AMP-bound structure. The structures as a whole are rendered semi-transparently. Boxes highlight regions with important changes, and key features are rendered in fully-opaque colors. (**d**) Close up of the upper boxed region in (**c**) illustrating pronounced changes in the positions of β-Phe405 and β-Ser391 upon binding of nucleotide. (**e**) Detailed representation of the lower boxed region in (**c**). A cross-subunit β-Asn578/α-Glu85 interaction in the ligand-free structure is disrupted following AMP binding. This allows α-Glu85 to move in the direction of the arrow and leads to destabilization of the Mn-proximal α-helix shown in (**a**). These changes together allow the α-Glu89 side chain to rotate to coordinate to the Mn.

In the absence of bound substrates or nucleotides (ligand-free form), the AC structure has an open cleft at the α/β subunit interface leading to the nucleotide-binding site in the β-subunit (Fig. 2a). Acetone and bicarbonate are presumed to be activated sequentially at the lone nucleotide-binding site^9^. Prior data are most consistent with the activation of acetone by ATP to generate a phosphoenolacetone intermediate and ADP that then reacts with bicarbonate to generate carboxyphosphate and AMP^17^. One or both phosphorylated intermediates are proposed to coordinate to the Mn site, forming the new C-C bond of acetoacetate while generating two phosphate leaving groups. However, the structure of the native AC shows the nucleotide binding site located ~40 Å from the Mn site of acetoacetate formation, with no visible pathway for the transfer of reactive phosphorylated intermediates. This is furthermore surprising since previous electron paramagnetic resonance (EPR) studies on the *Rhodobacter capsulatus* AC indicated that different nucleotide-bound states of AC exhibited markedly different EPR signatures^12^. This was interpreted as evidence that the nucleotide interacted directly with the Mn.

The apparent inconsistencies between the spectroscopic and structural work described here can be rationalized by comparing the AMP-bound and ligand-free structural states of AC. The AMP-bound structure exhibits dramatic rearrangements of the β-subunits relative to the ligand-free state, with a C_α_ RMSD difference of 6.3 Å, while the α- and γ-subunits remain almost the same with C_α_ RMSD of 1.3 Å and 0.3 Å, respectively. The rearrangements in the β-subunits result in several differences with the native, ligand-free structure. First, in the AMP-bound enzyme, the displacement (11–18 Å) of several amino acid residues that participate in nucleotide interactions leads to closing of the substrate-access channel (Fig. 2c). Most notably, β-Phe405 is displaced by 16 Å, to a new position where it interacts with the adenine of AMP through π-stacking, capping off the access channel. β-Ser391 and the helix of which it is a part rotate into hydrogen bonding distance with the ribosyl moiety (Fig. 2d). Second, a new internal channel opens up in the AMP-bound structure, connecting the nucleotide-binding site and the Mn site where acetone carboxylation occurs (Fig. 2b). This internal channel in AC is reminiscent of the channel observed in the structure of carbamoyl phosphate synthetase, which was proposed to protect the phosphorylated intermediate (carboxyphosphate) from bulk solvent where it would be rapidly hydrolyzed^18^. An internal channel could serve an analogous role here toward the same intermediate, and could steer both phosphorylated intermediate species toward the Mn site at the end of the channel. Third, the Mn site undergoes substantial changes in coordination and substrate accessibility in the nucleotide-bound state. The ligand-free form of AC contains α-Glu89 positioned at the C-terminus of a Mn-proximal α-helix (residues α82–87) which blocks access to the Mn active site from the α/β interface (Fig. 2a). After nucleotide binding, conformational changes within the β subunit result in loss of an interaction between the side chains of β-Asn578 and α-Glu85 and formation of a new one between β-Asn578 and the carbonyl of β-Met571 (Fig. 2e). The Mn-proximal α-helix concurrently forms a disordered loop (α-81–87), allowing the opening of the substrate channel and the bidentate coordination of the α-Glu89 carboxylate to Mn (Fig. 2e). These observed changes in Mn coordination suggest a means of rationalizing the previous EPR studies showing changes in the Mn spectra in response to nucleotide binding^12^. Though the nucleotide does not interact directly with the metal, the conformational shifts that occur along with nucleotide binding result in substantial changes in the Mn coordination environment, which could in turn influence the EPR spectra.

**Figure 3:**
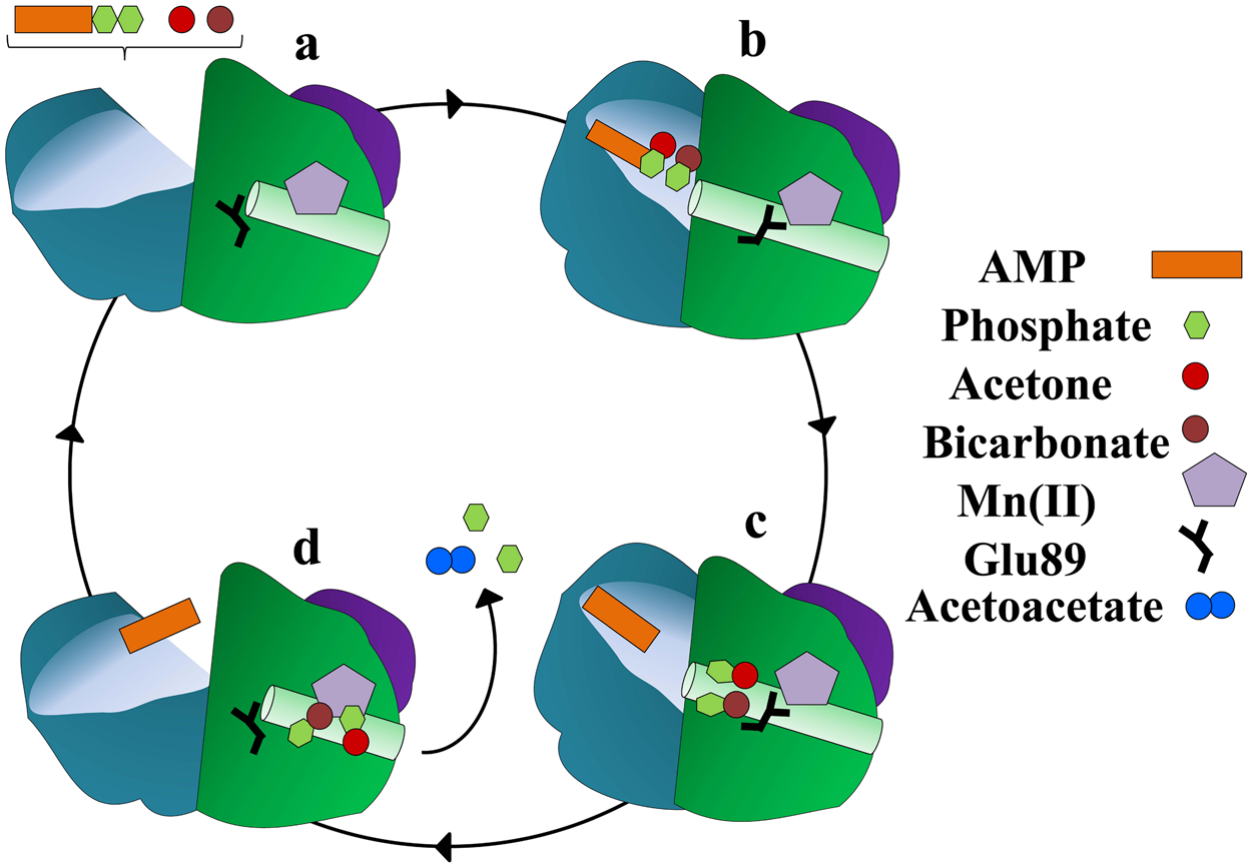
Substrate channel cycle. (**a**) The nucleotide and carbon substrates (acetone and bicarbonate) travel to the binding site through the substrate channel that is observable in the ligand-free AC structure with an opening near the α/β subunit interface. (**b**) Binding of substrates and the subsequent  phosphorylation reactions presumably start the cycle by closing off the substrate channel, trapping nucleotide and possibly the two reactive intermediates inside, and opens a new channel within the α-subunit’s interior. This interior channel, which is easily identifiable in the AMP-bound structure, connects the site at which acetone and HCO_3_^−^ are phosphorylated with the Mn active site 40 Å away. Glu89 shifts into a Mn-coordinating position when the interior channel opens. (**c**) The AMP bound structure, which now has a hexacoordinate Mn, represents the enzyme in the state encountered by the carboxy- and acetone-phosphates. (**d**) Our acetate/AMP bound structure describes two intermediates or possibly the acetoacetate product electrostatically displacing Glu89, which results in the formation of the Mn-proximal α-helix. This switch triggers the closing of the interior channel and the reopening of the substrate channel for nucleotide departure and new access. Acetoacetate may then exit through the dimer interface.

**Figure 4:**
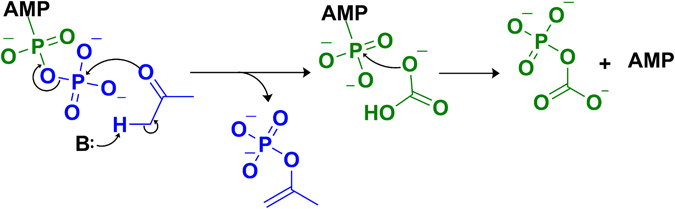
Phosphorylation of substrates. The phosphorylation reaction takes place in the β-subunit nucleotide-binding site. The phosphorylation of acetone starts with deprotonation of α-carbon of acetone by a yet unidentified basic residue, forming an enolate which is phosphorylated by the γ-phosphate (blue) to form phosphoenolacetone. Bicarbonate is subsequently phosphorylated by the β-phosphate (green) to form carboxyphosphate and AMP.

**Figure 5:**
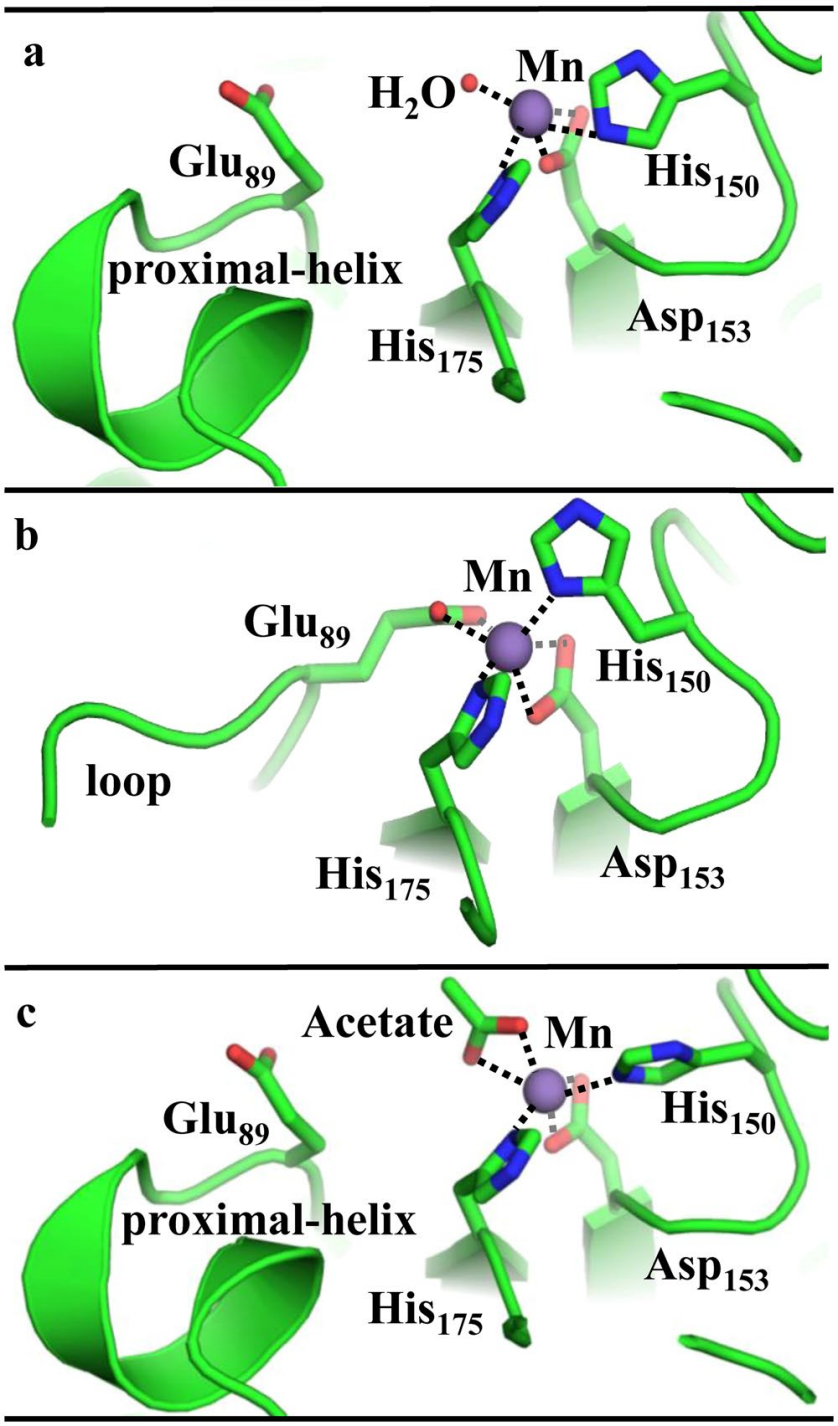
Conformational changes enforce changes in coordinating residues in the Mn active site. **(a**) The ligand-free structure shows Mn coordinated to a water, His150, His175, and Asp153 (all deriving from the α-subunit). The Mn-proximal α-helix is structured, blocking the Mn site from access to the α/β interface and solvent. (**b**) The AMP-bound structure shows Mn coordinated to His150, His175, Asp153, and Glu89. Nucleotide binding to the β subunit induces large conformational changes that cause the Mn-proximal α-helix to form a disordered loop, allowing the Glu89 side chain to coordinate to the Mn site. (**c**) Acetate-bound structure shows the displacement of Glu89 and the concomitant reorganization of the Mn-proximal α-helix. The internal channel is closed and the substrate-access channel is open in this structure, which is proposed to illustrate the effects of displacement of Glu89 from the Mn by phosphorylated intermediates.

The ligand-free and AMP-bound structures, in conjunction with a third, acetate- and AMP-bound structure representing the AC-product complex and described below, provide a strong basis for inferring an overall mechanism for ATP dependent carboxylation of acetone (Fig. 3). The ligand-free structure is assigned as the resting state awaiting the binding of substrates ATP, acetone, and bicarbonate at the nucleotide-binding site. Substrates may access this site at the substrate-binding cleft where phosphorylation of acetone, likely following its deprotonation (p*K*a = 20)^19^, is followed in turn by the phosphorylation of bicarbonate to form AMP, phosphoenolacetone, and carboxyphosphate (Fig. 4). The structure of AC in the presence of bound AMP and in the absence of these phosphorylated intermediates may therefore represent a trapped state, bound to the product AMP but lacking intermediates needed to proceed further in the reaction. This trapped state reveals the internal channel that is proposed to protect the semistable phosphorylated intermediates from the bulk solvent as they travel from the nucleotide-binding site to the Mn site (Fig. 2b). The Mn in this structure appears to be coordinately saturated, with α-His150 and α-His175 as monodentate ligands and two side chains each bound in a bidentate mode (α-Asp153, α-Glu89) (Fig. 5b). This state may best represent the coordination environment encountered by the phosphoenol acetone and carboxyphosphate intermediates during catalysis. Upon binding of the intermediates the side chain of α-Glu89 could potentially be displaced, allowing one or both to coordinate to the Mn. The sidechain of α-Asp153 could likewise shift from a bi- to a mono-dentate coordination mode, allowing the Mn to coordinate and activate an attacking water molecule.

**Figure 6:**
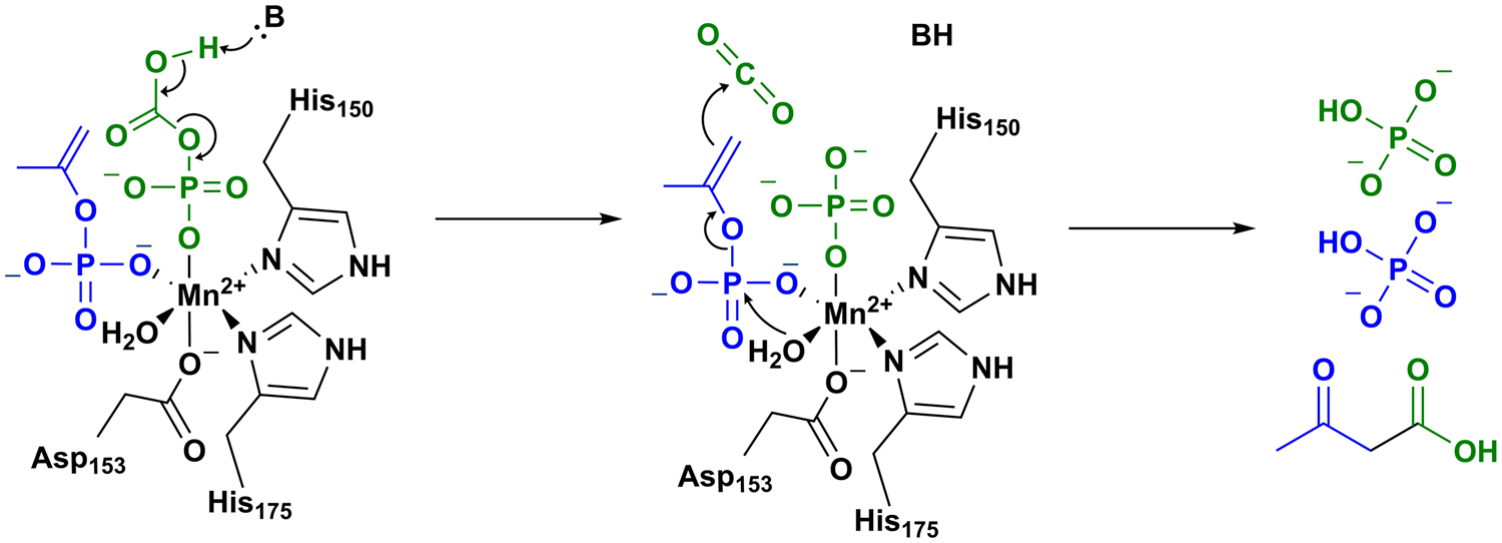
Reaction of intermediates to form products. A Mn active site mechanism of action is proposed. At the Mn active site, we propose a mechanism of action in two steps. It is reasonable to believe from our acetate-bound structure that the Mn is responsible for the ordering of the intermediates. (**A**) First, a residue near the carboxyl side of the carboxyphosphate will deprotonate the carboxylic acid which triggers a decarboxylation event to produce CO_2_ and an inorganic phosphate. This initial reaction contains similarities to the PEPC Mn active site where a histidine residue stabilizes the carboxyphosphate intermediate, the AC-acetate bound structure shows His111 in proximity to the active site and can serve an analogous role. (**B**) After the decarboxylation event, the enol-acetone will then bond with the carbon dioxide after hydrolysis of its phosphate group. (**C**) The products of the reaction acetoacetate and two inorganic phosphates will then exit through the dimer interface restarting the Mn active site.

To examine the proposed ligand exchange mechanism at the Mn catalytic site, the acetate/AMP-bound structure of AC was characterized. Acetate was used as a product analog because of the instability of the native product acetoacetate in aqueous solution. In this structure, acetate is bound at the Mn site and α-Glu89 resumes its position oriented away from the Mn (Fig. 5c). The displacement of α-Glu89 by the phosphorylated intermediates immediately before their conversion to products could be part of a reciprocal conformation switch leading to the re-closing of the internal channel. The collapse of this channel is apparent in the acetate/AMP-bound structure (Fig. 5c), which shows a reorganized Mn-proximal α-helix in addition to movement of α-Glu89. Such collapse would prevent the diffusion of intermediates away from the Mn. Once the intermediates are bound at the Mn site, the decay of carboxyphosphate could be spontaneous or catalyzed by a nearby basic residue to produce CO_2_ and phosphate. Once CO_2_ has been produced, hydrolysis by a potentially Mn-activated water/hydroxide on the phosphoenolacetone would lead to the formation of an enolacetone intermediate and the second phosphate product. The electron-rich enol double bond would then nucleophilically attack the CO_2_ derived from the decomposition of carboxyphosphate, forming acetoacetate (Fig. 6).

Well-described carboxylases such as acetyl-CoA carboxylase (ACC) and phosphoenolpyruvate carboxylase (PEPC) function by transforming the carboxylation substrate into a highly reactive carbanion that is resonance-stabilized as an enolate or enolate adduct^2^. The enolate subsequently attacks the electron-poor carbon of CO_2_ to form a new C-C bond. Some carboxylases react with bicarbonate rather than directly with CO_2_^20^. HCO_3_^−^ is dehydrated in an energy- (ATP or ADP) consuming reaction where the HCO_3_^−^ is, at the same time, activated by phosphorylation (−49 kJ/mol per phosphoryl bond hydrolyzed)^21^. The resulting carboxyphosphate is highly unstable, breaking down to yield CO_2_ and inorganic phosphate within 70 ms under physiological conditions^22^. The biotin cofactor then captures the released CO_2_ as N1-carboxybiotin. The carboxylate group can subsequently be transferred to an attacking enolate substrate^20^.

Biotin-dependent carboxylases such as ACC are often complex enzymes, consisting of one or more protein subunits that separately activate and temporally coordinate the joining of substrates in a new C-C bond^23^. HCO_3_^−^ utilizing phosphoenolpyruvate carboxylase (PEPC) is both biotin- and ATP-independent^24^. These enzymes are simpler than their biotin-containing counterparts, possessing a single Mn at the active site. The phosphoryl group is transferred from the already phosphorylated PEP to HCO_3_^−^, releasing water and generating a highly reactive carboxyphosphate and the pyruvate enolate anion adjacent to one another. These are proposed to coordinate Mn before reacting to yield oxaloacetate and phosphate^24^.

The structurally-inferred mechanism for AC presented here suggests a new carboxylation mechanism that has features in common with the ACC and PEPC paradigms. Like ACC, acetone carboxylase is an ATP- and bicarbonate-dependent multisubunit enzyme with a ketone as the carboxylation substrate. Like PEPC, acetone carboxylase lacks biotin but has a catalytically essential Mn center around which the phosphorylated intermediates may couple, forming a new C-C bond. Distinct from both ACC and PEPC, acetone carboxylase sequentially activates *both* of its substrates by phosphorylation. The fact that the phosphoenol pyruvate substrate arrives at PEPC already phosphorylated (by phosphoenolpyruate synthase) may reflect the much greater stability of this activated species, relative to the phosphoenol acetone intermediate in the AC reaction^25^. Activating both substrates in the same enzyme protects the two unstable phosphorylated intermediates within the interior of AC, and costs the enzyme two high-energy phosphoryl bonds (−98 kJ/mol). This energy input exceeds the 34.8+/−8.1 kJ/mol required for the formation of acetoacetate form acetone and bicarbonate but is potentially important for driving the large observed structural transitions in the β-subunits of AC^26^. These transitions appear to be essential for incorporating all 3 substrates into the protein, and then for retaining the phosphorylated intermediates in a solvent-restricted, enclosed space where they can access the Mn^27^. The fact that AC catalyzes an ADP-accumulating reaction between ATP and acetone suggests that this reaction occurs first^9^, and may occur via the initial deprotonation of the acetone adjacent to the γ-phosphoryl of ATP. Transfer of a second phosphoryl group, from ADP to bicarbonate, would then generate the carboxyphosphate intermediate.

In the structure of PEPC, a dichlorinated phosphoenolpyruvate substrate analog coordinates the Mn^28^. It appears likely that HCO_3_^−^ also coordinates to the metal in PEPC, and that proximity between the substrates is important for facilitating the phosphoryl transfer between the two. The Mn center in AC could, by analogy, coordinate the phosphoenolacetone and/or carboxyphosphate intermediate. In order to interact with the Mn, the carboxyphosphate intermediate would have to travel 40 Å within its ~70 ms lifetime. Though this is a relatively long distance, the necessary speed is well below that implied by the approximate size of the inner channel and the second order rate constant for a diffusion controlled reaction under aqueous conditions (7.4 × 10 ^6^ M^−1^ s^−1^, 25 °C)^29^. Facilitating the joining step between carboxyphosphate and phosphoenolacetone, by organizing the two in space and possibly by stabilizing their negative charges, is one possible role for Mn. Another might be to act as a Lewis acid toward the water molecule that displaces the phosphate from the phosphoenolacetone intermediate.

For the structurally related APC it was proposed that a large-scale conformational change allowed the sites for acetophenone phosphorylation and carboxylation to be in close proximity to one another^11^. This was proposed on the basis of the structure of a single state in which the sites are separated as in the case of AC by nearly 40 Å. For AC the ability to capture several states for structural characterization reveals an overall mechanism of how the two sites communicate over this distance and how substrates travel between the sites. Given the structural similarity between APC and AC it is seems likely that the two enzymes have conserved mechanisms for communicating between the sites but this will have to be resolved with the determination of additional mechanistically relevant states of APC.

Traditionally, establishing the mechanism of carboxylating enzymes has been very complicated largely due to the experimental difficulties associated with working with gaseous CO_2_ itself, as a substrate that disproportionates in aqueous solution to multiple species. Carboxylation chemistry is, however, of paramount and growing importance as a result of the continued rise of environmental CO_2_ concentrations and the associated implications for global climate change. An elegant mechanism for AC in which a single ATP is used to activate both the organic substrate acetone and bicarbonate in an obligatorily sequential and stoichiometrically balanced manner has emerged from a long series of biochemical data. Capturing AC in multiple structural states here has revealed large conformational changes that occur during catalysis, which presumably protect highly reactive intermediates phosphoenolacetone and carboxyphosphate from degradation in aqueous solvent consistent with what has been proposed for the related APC. However, clearly distinct from the proposed reaction cycle for APC, the elegant coupling of the conformational change of AC to changes in the coordination environment of the active site Mn where acetoacetate forms provide a structural rationale for the joining of the two phosphorylated intermediates in AC. The results presented here reveal new and unique elements of carboxylation chemistry that could be utilized in biological or bioinspired catalytic strategies for the conversion of CO_2_ into chemical feedstock and/or biomass.

## Methods

### Cloning of acetone carboxylase genes from *X. autotrophicus* Py2 and construction of expression system

The three genes coding for the AC from *X. autotrophicus* Py2, *acxA* (Xaut_3509), *acxB* (Xaut_3510), and *acxC* (Xaut_3511), were amplified by PCR using *X. autotrophicus* Py2 genomic DNA and cloned independently in pET-Duet system vectors (EMD Millipore, Merck, Darmstadt, Germany). The *acxA* gene encoding the β subunit *(*AAL17710.1) was amplified by two successive PCR reactions using the following primers pairs: (1) (Forward) 5′-TCACCACAGCAGCGGC **ATG**AACGTTCCCGTGGGACACCTG-3′ and (Reverse) 5′-CCG*CTCGAG* **TCA**CACCTCGCGCAGGTGGAACA-3′; (2) (Forward) 5′-GGAATTC*CAT* ***ATG*** CATCACCATCATCACCACAGCAGCGGCATGAACGTT-3′ and (Reverse) 5′-CCG*CTCGAG* **TCA**CACCTCGCGCAGGTGGAACA-3′. NdeI and XhoI sites (in italics above) were, respectively, inserted upstream and downstream of the start and the stop codons (in bold), and a (His)_6_-tag coding sequence (underlined) was inserted directly upstream of the start codon of the open reading frame. NdeI and XhoI sites were used to insert *acxA* into the multiple cloning site 2 (MCS2) of the RSFDuet vector (conferring kanamycin resistance). The *acxB* gene encoding the α subunit (AAL17711.1), was amplified by PCR using the following primers: (Forward) 5′-GGAATTC*CAT* ***ATG***AACGTGACTGTTGACCAGAGCA-3′ and (Reverse) 5′-CCG*CTCGAG* **TCA**TTCCTCCACGAACTGCA-3′. NdeI and XhoI sites (in italics above) were, respectively, inserted upstream and downstream of the start and the stop codons (in bold) of the open reading frame and used to clone the *acxB* gene in the MCS2 of the pET-Duet vector (conferring ampicillin resistance). The *acxC* gene encoding the γ subunit (AAL17712.1) was amplified by PCR using the following primers: (Forward) 5′-GGAATTC*CAT* ***ATG***GCCTATACCCGCTCGAAGATCGTCGA-3′ and (Reverse) 5′-CCG*CTCGAG* **TCA**GGCGTCGGCCCGCT-3′. NdeI and XhoI sites (in italics above) were, respectively, inserted upstream and downstream of the start and the stop codons of the open reading frame and used to insert the *acxC* gene in the MCS1 of the CDFDuet vector (conferring streptomycin resistance). The resulting plasmids (RSFDuet-*acxA*, pETDuet-*acxB*, CDFDuet-*acxC*) were sequenced and further used to co-transform *Escherichia coli* (BL21DE3) (EMD Millipore) for heterologous expression of *X. autotrophicus* Py2 AC. *E. coli* cells were grown on Luria broth (LB) medium supplemented with 50 µM MnCl_2_ and appropriate antibiotics: ampicillin (100 μg/mL), kanamycin (50 μg/mL), streptomycin (25 μg/mL).

### Production and purification of recombinant His-tagged acetone carboxylase from *X. autotrophicus* Py2

The recombinant *E. coli* (BL21DE3) strain overexpressing AC was grown in LB in both 10 L fermenters and 2.5 L shaker flasks using auto-induction media^30^. Cells were also grown in 2.5 L shaker flasks with auto-induction media supplemented with selenomethionine (62.5 mg) for structure determining purposes. Cells were collected by centrifugation 7,000 × *g* for 10 min and stored at −20 °C until use. Cells were lysed by sonication in buffer containing 25 mM MOPS, 1 mM BME, 0.1 mM EDTA, 0.1 mM EGTA, and 20% glycerol, pH 7.6. Cell-free extracts were prepared by centrifuging at 100,000 × *g* for 30 min to remove particulates and membrane fragments. The resulting supernatant was loaded onto a 5 mL His-NTA column and eluted with a gradient of imidazole (25 mM MOPS, 1 mM BME, 0.1 mM EDTA, 0.1 mM EGTA, 20% glycerol, 0–400 mM imidazole, pH 7.6). Fractions containing acetone carboxylase were pooled and concentrated using Amicon Ultra-4 ultra-filtration centrifugal filters (EMD Millipore). The concentrated protein was then desalted on a P2 column (GE Healthcare Life Science) with 25 mM MOPS, 200 mM NaCl, pH 7.6. Fractions were pooled and stored at −80 °C until further use.

### Production and purification of native acetone carboxylase from *X. autotrophicus*

*X. autotrophicus* 7C^T^ (DSM-432) cells were obtained from the Deutsche Sammlung von Mikroorganismen und Zellkulturen (DSMZ). *X. autotrophicus* cells were grown 3 L at a time in 1 L batches of DSMZ-260 mineral media (DSMZ, Germany) containing 13.6 mM sodium pyruvate and 40 mM acetone at 30 °C, shaking at 220 rpm until they reached an optical density of approximately 4.0 at 600 nm. Cells were harvested by centrifugation at 4 °C for 30 min at 5000 × *g*. Pellets were resuspended in 12.5 mM MOPS, pH 7.6, 0.1 mM EDTA, 0.1 mM EGTA, 20% (v/v) glycerol, and lysed using a sonicator (Sonics Vibracell Ultrasonic Processor, 6 mm tip) for 5 minutes with 2-second pulses at 50% amplitude. The lysate was centrifuged at 5000 × *g* for 30 min at 4 °C to remove the cellular debris. The supernatant was diluted with the same buffer (12.5 mM MOPS, pH 7.6, 0.1 mM EDTA, 0.1 mM EGTA, 20% glycerol), and filtered. The diluted sample (1 L) was applied to the conditioned DEAE anion exchange column (20 mL) overnight, which was previously equilibrated with 12.5 mM MOPS, pH 7.6, 0.1 mM EDTA, 0.1 mM EGTA, 20% glycerol. The protein was eluted using a linear gradient of KCl (from 27 to 135 mM). The eluted fractions were pooled and diluted with 1.5 M ammonium sulfate, 12.5 mM MOPS, pH 7.6, 0.1 mM EDTA, 0.1 mM EGTA, 20% glycerol up to 1 L to use for hydrophobic interaction chromatography. The diluted protein sample (1 L) was loaded onto a 5 mL phenyl sepharose column overnight, and eluted using a linear gradient of the resuspension buffer with ammonium sulfate (1.5 to 0 M). Lastly, the eluted fractions from the phenyl sepharose column were pooled. Native AC was isolated in polished form on a Superdex-200 HiLoad 16/60 gel filtration column in 12.5 mM MOPS, pH 7.6, 0.1 mM EDTA, 0.1 mM EGTA, 10% glycerol, 108 mM KCl. The purified protein was concentrated to ~15 mg/mL for crystallization.

### Enzyme activity assays

The activity of the purified recombinant acetone carboxylase from *X. autotrophicus* Py2 enzyme was determined using a routine assay of acetone carboxylase which is based on continuous spectrophotometric assays^8, 17^. The acetone carboxylase activity of the purified recombinant acetone carboxylase enzyme from *X. autotrophicus* Py2 was coupled to the activity of 3-hydroxybutyrate dehydrogenase confirming the formation of acetoacetate as the product of acetone carboxylation.

### Structure determination, refinement, and analysis

Recombinant AC crystals were obtained by the hanging-drop vapor diffusion method at 25 °C using 7–14 mg/ml of AC protein with 14–18% PEG 3350 as the precipitant in 0.2 M MgSO_4_, pH 6.3. Nucleotide additives were added to the crystal before cyro-protectant at a final concertation of 5 mM. The data were collected from flash-cooled crystals (protected by well condition plus 15% v/v glycerol) with a continuous flow of liquid nitrogen at 100 K on BL12-2 (SLAC National Accelerator Laboratory), and at 19ID-SBC beamline (Argonne National Laboratory). The diffraction images were indexed, integrated and scaled using HKL2000^31^. The initial structure of the recombinant protein was solved to 2.6 Å by single anomalous dispersion (SAD) using selenium as anomalous scatterer (Table S1) and using the Phenix suite of programs (HYSS, Phaser, and Resolve) as implemented in Autobuild wizard^32^. The Autobuild run identified all 102 Se sites, allowing non-crystallographic symmetry (NCS) operators to be found. An inclusion of the two-fold NCS in density modification resulted in improvement of the experimental phases from the initial figure of merit (FOM) of 0.34 to the final FOM of 0.70 and consequently led to high quality, interpretable density maps. The initial automated model building yielded a ~60% completed model with R/Rfree = 038/0.44. Several rounds of manual rebuilding with the program COOT^33^ completed the model to a final R/Rfree to 19%/22%.

The AMP and acetate-bound crystals of native AC from *X. autotrophicus* 7 C were obtained by hanging drop vapor diffusion and using 3.5 mg/ml protein with 10 mM ATP and 10 mM acetone at 17 °C with reservoir 0.3 M calcium acetate hydrate, pH 7.5, 30% (w/v) PEG 3350 with drops containing this solution and an equal volume of protein solution. Crystals were cryoprotected by soaking in the mother liquor with 30% volume PEG 400 added. X-ray diffraction data were collected at 100 K at beamline I04-1, Diamond Light Source, UK. The data were processed and scaled with xia2^34^ using DIALS^35^. The structure was solved by molecular replacement using Phaser^31^ with the recombinant ligand-free AC structure as the model. The sequence was the same as the Py2 strain except for a six amino acid changes observed in the density and supported by sequence alignments of homologues (Table S2). Two (αβγ)_2_ dimers were found in the P1 unit cell, with AMP bound and no acetone visible in the density. The solutions were refined and improved using phenix.refine^32^ with cycles of rebuilding in COOT^33^ to a final R/Rfree to 19%/22% for AMP-bound AC, 20%/25% ligand free AC, and 19.2%/21.7% for AMP and acetate bound native AC structures. The presence of Zn and Mn, and the absence of Fe metals were identified by x-ray fluorescence spectra and further confirmed by the collection of anomalous dispersion data. Data collection and final refinement statistics are given in supporting information section (Table S1). Molecular figures were prepared using PyMol^36^ (http://www.pymol.org) and the Caver^37^ plugin (http://www.caver.cz/).

### Anomalous data determination

Beamline I03 (Diamond Light Source, UK) was used to collect anomalous determination data and was processed with xia2^34^ and DIALS^35^. For the natively purified protein, we calculated element specific maps, to look at the natural *in vivo* metal content of the enzyme. Element-specific maps were calculated by the technique of collecting datasets above and below the absorption K-edge. The anomalous differences (DANO), were calculated for each dataset, and scaled together with scaleit^38^, and the difference between DANO sets calculated. In combination with model phases, these double differences were used to calculate an element-specific anomalous double-difference map^39, 40^. This was then 4-fold NCS averaged in the P1 unit cell of the native crystals for each chain. Each pair of these anomalous datasets was collected from a different region of a long rod-shaped natively purified AC crystal with the below-edge dataset collected first to avoid signal artifacts due to radiation damage. Each dataset was collected 25 eV above or below the relevant elemental K-edge. Data statistics are shown in Table S2, and the scaled datasets are available as supplemental files: zn_above_below_scaleit1__DDANO.mtz mn_above_below_scaleit1__DDANO.mtz fe_above_below_scaleit1__DDANO.mtz.

### Data Availability

The PDB submission codes for AC structures of AMP-bound, AMP-Acetate-bound and ligand free forms were deposited in the PBD databank with codes 5SVB, 5M45, and 5SVC, respectively.

## Electronic supplementary material

Supplementary Information
