# Supplementary material for: Structural Basis for the Mechanism of ATP-Dependent Acetone Carboxylation
Source: Sci Rep. 2017 Aug 3;7:7234. doi: 10.1038/s41598-017-06973-8 (PMC5543143; doi:10.1038/s41598-017-06973-8)
Supplement: Supplementary file 1 — Supplementary Information [file 41598_2017_6973_MOESM1_ESM.docx]

**Supplementary Information**

**Structural Basis for the Mechanism of ATP-Dependent Acetone Carboxylation**

Florence Mus, Brian J. Eilers, Alexander B. Alleman, Burak V. Kabasakal, Jennifer N. Wells, James W. Murray, Boguslaw P. Nocek, Jennifer L. DuBois, John W. Peters

**Table S1. Data collection and refinement statistics.**

|  | AMP bound (Se peak) | Native Acetate and AMP bound | Ligand free |
| --- | --- | --- | --- |
| **Data collection** |  |  |  |
| PDB ID | 5SVB | 5M45 | 5SVC |
| Wavelength (Å) | 0.979 | 0.928 | 0.979 |
| Space group | *P*212121 | *P*1 | *P*21 |
| Cell dimensions |  |  |  |
| *a*, *b*, *c* (Å) | 89.928,100.209,441.384 | 86.915, 39.733,165.827 | 76.76,265.159,122.170 |
|   γ () | 90, 90, 90 | 65.895, 86.537,8.686 | 90, 92.33, 90 |
| Resolution (Å) | 39.62-2.65(2.7-2.65) | 126-1.87 (1.90-1.87) | 39-2.7(2.73-2.70) |
| *R*merge | 0.146 (1.01) | 0.114 (0.887) | 0.113 (0.827) |
| *I* / *I* | 13.2 (1.8) | 7.0 (1.2) | 10.2 (1.8) |
| Completeness (%) | 98.3 (97.8) | 97.8 (96.8) | 93.0 (93.4) |
| Redundancy | 6.5 (5.8) | 3.5 (3.3) | 4.7 (4.6) |
|  |  |  |  |
| **Refinement** |  |  |  |
| Resolution (Å) | 2.64 | 1.87 | 2.7 |
| No. reflections | 112,445 | 544,134 | 124,636 |
| *R*work / *R*free | 0.198/0.227 | 0.192/0.217 | 0.201/0.255 |
| No. atoms | 25,817 | 56,067 | 25,811 |
| Protein | 25,440 | 51,312 | 25,694 |
| Ligand/ion | 60 | 192 | 4 |
| Water | 317 | 4511 | 111 |
| *B*-factors |  |  |  |
| Protein | 43.2 | 15.6 | 44.98 |
| Ligand/ion | 66.25 | 30 | 55.6 |
| Water | 37.9 | 45.0 | 51.5 |
| R.m.s deviations |  |  |  |
| Bond lengths (Å) | 0.003 | 0.014 | 0.011 |
| Bond angles () | 0.518 | 1.588 | 1.156 |

*Highest resolution shell is shown in parenthesis.

* Values in parentheses are for the highest resolution shell.

**Table S2. Anomalous data collection statistics.**

|  | Fe-below K-edge | Fe above K-edge | Mn-below K-edge | Mn-above K-edge | Zn-below K-edge | Zn-above K-edge |
| --- | --- | --- | --- | --- | --- | --- |
| wavelength (energy) | 1.74946 Å  (7087.0 eV) | 1.73720 Å  (7137.0 eV) | 1.88885 Å  (6564.0 eV) | 1.90335 Å  (6514.0 eV) | 1.28698 Å  (9633.6 eV) | 1.28035 Å(9683.6 eV) |
| unit cell lengths (Å) | 86.19, 138.03,  164.08 | 86.41, 138.3,  164.39 | 85.88, 137.76,  163.78 | 85.83, 137.73,  163.82 | 85.84, 137.64,  163.68 | 85.94, 137.68,  163.82 |
| angles (º) | 65.89, 86.58,  88.85 | 65.89, 86.57,  88.87 | 65.87, 86.48,  88.82 | 65.87, 86.51,  88.84 | 65.81, 86.59,  88.86 | 65.80, 86.60,  88.89 |
| space group | P1 | P1 | P1 | P1 | P1 | P1 |
| Resolution range (Å) | 2.44-125.99  (2.44-2.48) | 2.85-126.26  (2.85-2.90) | 149.21-2.69  (2.73-2.69) | 149.26-2.59  (2.59-2.63) | 2.34-81.05 (2.34-  2.38) | 2.5-81.09 (2.40-  2.44) |
| Total Reflections | 1659956 (70629) | 1075571 (52005) | 1258563 (58227) | 1397642 (66559) | 1879900 (62718) | 1781500 (72736) |
| Unique  Reflections | 246751 (11398) | 156462 (7287) | 183122 (8732) | 204336 (9725) | 276512 (10149) | 260982 (11967) |
| R-merge (%) | 0.142 (1.112) | 0.187 (1.248) | 0.188 (1.082) | 0.185 (1.104) | 0.150 (0.915) | 0.161 (0.988) |
| I /σ (I) | 9.0 (1.5) | 7.8 (1.6) | 6.4 (1.4) | 7.1 (1.5) | 8.7 (1.6) | 7.9 (1.5) |
| Completeness (%) | 95.8 (88.2) | 97.0 (90.6) | 96.2 (92.5) | 95.9 (91.6) | 96.0 (70.8) | 96.0 (89.8) |
| Anomalous  Completeness (%) | 92.9 (4.2) | 94.4 (4.4) | 93.5 (4.4) | 93.1 (4.4) | 93.7 (3.4) | 95.2 (4.3) |
| Multiplicity | 6.7 (6.2) | 6.9 (7.1) | 6.9 (6.7) | 6.8 (6.8) | 6.8 (6.2) | 6.8 (6.1) |
| Anomalous  Multiplicity | 3.4 (3.2) | 3.5 (3.6) | 3.5 (3.4) | 3.5 (3.5) | 3.4 (3.2) | 3.5 (3.1) |
| CC 1/2 | 0.995 (0.654) | 0.991 (0.691) | 0.990 (0.727) | 0.992 (0.704) | 0.994 (0.668) | 0.993 (0.657) |
| anomalous correlation | 0.168 (0.015) | 0.269 (0.394) | 0.189 (0.017) | 0.108 (0.004) | 0.102 (-0.005) | 0.171 (0.016) |
| anomalous slope | 1.011 | 1.043 | 1.021 | 1.001 | 0.999 | 1.027 |
| R factor to below edge dataset |  | 0.124 (0.322) |  | 0.131 (0.351) |  | 0.137 (0.321) |

*Values in brackets refer to the high resolution shell.


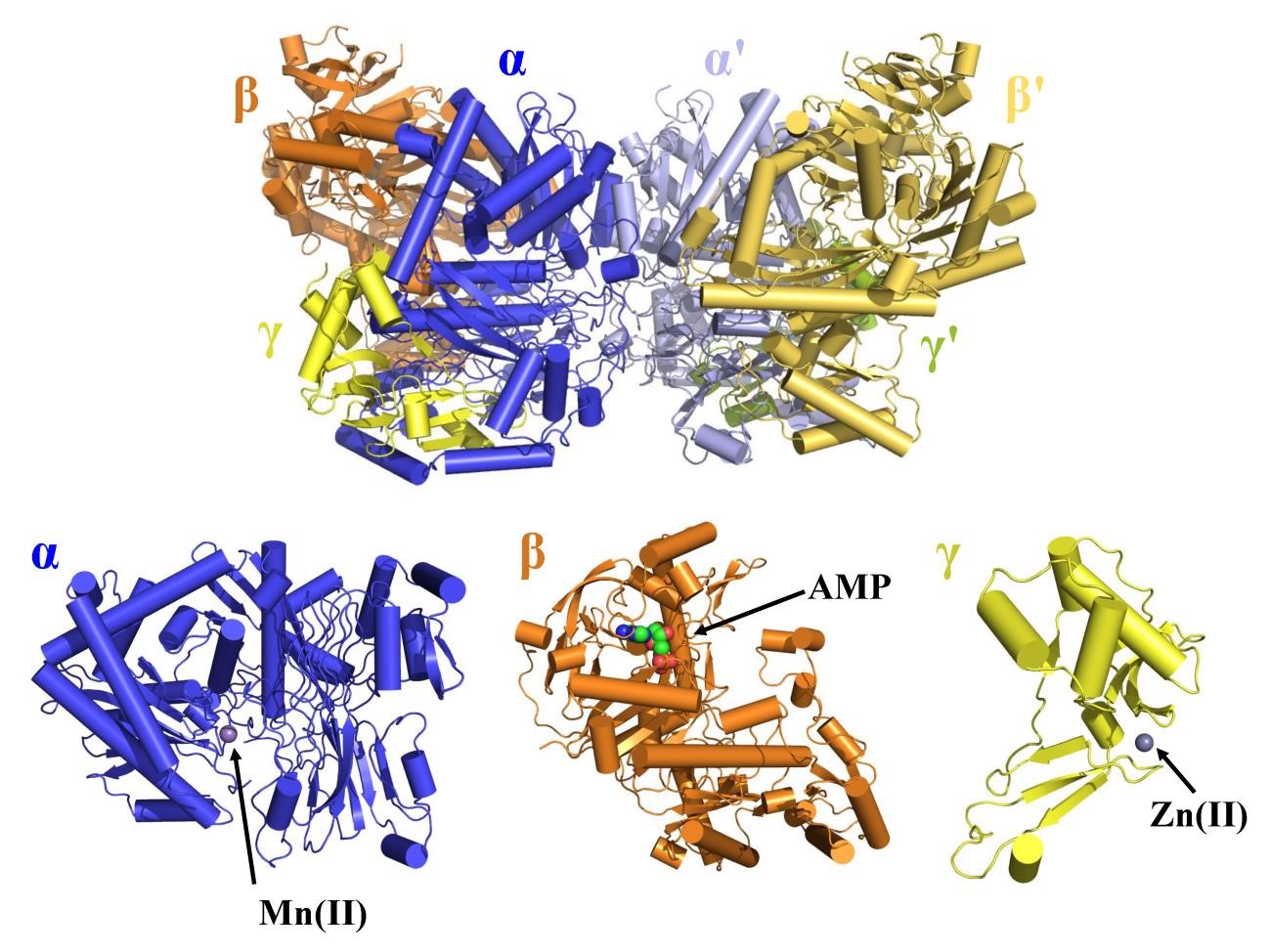


Figure S1. **Complete structure of heterohexamer AC.** Cylinder and sheet model shows connectivity of the α subunits (blue and light blue) to make the dimer interface. The α subunits contain a Mn(II) active site which faces the subunit interface of the β subunits (orange and yellow-orange). The γ subunits (yellow and splitpea) connect to both α and β subunits and contain the Zn(II) binding site.


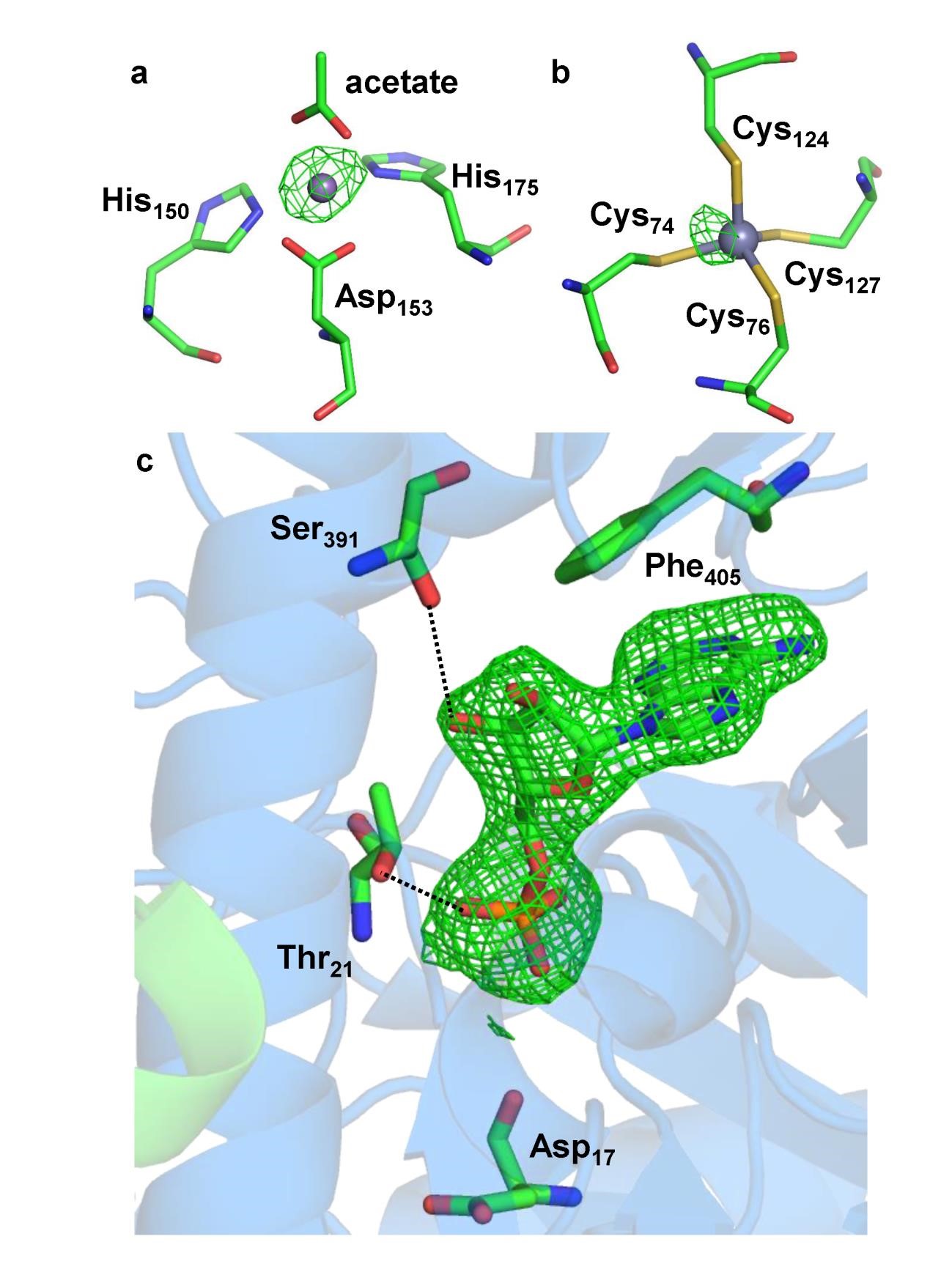


**Figure S2. Anomalous difference maps for Mn, Zn, and AMP sites in AC. (a)** anomalous difference map showing the Mn binding site for the AMP and acetate bound native AC structure.

Manganese NCS-averaged element-specific double-difference density contoured at 15 σ around the modelled manganese site in the alpha chain. **(b)** Anomalous difference map showing the Zn binding site for the AMP and acetate bound structure. Zinc NCS-averaged element-specific double-difference density contoured at 15 σ around four cysteines of the gamma subunit and the modelled zinc. No other peaks interpretable as zinc or manganese were observed. For the Fe double difference data, there were no interpretable peaks at any putative metal sites. From this it was concluded that, in the natively purified enzyme, the functional metal sites are Mn and Zn, with no detectable Fe in anomalous double-difference maps. **(c)** Detailed view of the AMP binding site for the AMP bound structure showing AMP omit difference density contoured at 3σ. Selected residues are shown as sticks. Residues Ser391, Thr21 form hydrogen bonds to the AMP molecule, Phe405 caps off the pocket and positions the adenosine ring. The backbone carbonyl of Asp17 is thought to interact with the gamma phosphate of ATP due to homology to other kinases.
